# *“We have to learn to cooperate with each other”:* a qualitative study to explore integration of traditional healers into the provision of HIV self-testing and tuberculosis screening in Eswatini

**DOI:** 10.1186/s12913-021-07323-1

**Published:** 2021-12-06

**Authors:** Bernadette Schausberger, Nqobile Mmema, Velibanti Dlamini, Lenhle Dube, Aung Aung, Bernhard Kerschberger, Iza Ciglenecki, Debrah Vambe, Esther Mukooza, Alison Wringe

**Affiliations:** 1 Médecins sans Frontières, Mbabane, Eswatini; 2 Eswatini National AIDS Program, Mbabane, Eswatini; 3 Médecins sans Frontières, Geneva, Switzerland; 4 National TB Control Program, Manzini, Eswatini; 5 Department of Population Health, London School of Hygiene and Tropical Medicine, Keppel St, London, UK

**Keywords:** Traditional healers, HIV self-testing, Tuberculosis screening, Health-seeking behaviour, Eswatini

## Abstract

**Background:**

Traditional healing plays an important role in healthcare in Eswatini, and innovative collaborations with traditional healers may enable hard-to-reach men to access HIV and tuberculosis diagnostic services. This study explored attitudes towards integration of traditional healers into the provision of HIV self-testing kits and sputum collection containers.

**Methods:**

A qualitative study was conducted in 2019–2020 in Shiselweni region, Eswatini. Eight male traditional healers were trained on HIV and tuberculosis care including distribution of HIV self-testing kits and sputum collection containers. Attitudes towards the intervention were elicited through in-depth interviews with the eight traditional healers, ten clients, five healthcare workers and seven focus group discussions with community members. Interviews and group discussions were conducted in SiSwati, audio-recorded, translated and transcribed into English. Data were coded inductively and analysed thematically.

**Results:**

81 HIV self-testing kits and 24 sputum collection containers were distributed by the healers to 99 clients, with 14% of participants reporting a reactive HIV self-test result. The distribution of sputum containers did not result in any tuberculosis diagnoses, as samples were refused at health centres.

Traditional healers perceived themselves as important healthcare providers, and after training, were willing and able to distribute HIV self-test kits and sputum containers to clients. Many saw themselves as peers who could address barriers to health-seeking among Swazi men that reflected hegemonic masculinities and patriarchal attitudes. Traditional healers were considered to provide services that were private, flexible, efficient and non-judgemental, although some clients and community members expressed concerns over confidentiality breaches. Attitudes among health workers were mixed, with some calling for greater collaboration with traditional healers and others expressing doubts about their potential role in promoting HIV and tuberculosis services. Specifically, many health workers did not accept sputum samples collected outside health facilities.

**Conclusions:**

Offering HIV self-testing kits and sputum containers through traditional healers led to high HIV yields, but no TB diagnoses. The intervention was appreciated by healers’ clients, due to the cultural literacy of traditional healers and practical considerations. Scaling-up this approach could bridge testing gaps if traditional healers are supported, but procedures for receiving sputum samples at health facilities need further strengthening.

## Introduction

Eswatini remains a country heavily affected by dual HIV and tuberculosis (TB) epidemics. By 2019, 27% of the adult population were living with HIV, while TB incidence was 389 per 100,000 persons [1, 2] with 70% of TB cases co-infected with HIV [3]. Major progress has been made in providing HIV services, with an estimated 95% of the 210,000 people living with HIV (PLHIV) knowing their HIV status, and among those diagnosed, 95% accessing antiretroviral therapy, although these proportions have remained consistently lower among men [4].

Reaching the remaining undiagnosed men living with HIV with testing services has proved particularly challenging in Eswatini, with barriers including stigma associated with being seen in HIV clinics, discomfort in dealing with female health providers due to dominant notions of masculinity, and a lack of trust in biomedical health facilities [5, 6]. Similarly, the country has reported sub-optimal TB notification rates [1], with the majority of “missing cases” being among men, further contributing to ongoing transmission and undermining the national aim of achieving epidemic control of both HIV and TB [1]. Barriers to care-seeking for TB by men hold many parallels to those for HIV, with both being highly stigmatised conditions [7]. For both infections, men may avoid seeking diagnoses because of the anticipated rupture to their social life and masculine identity, which includes being able to provide for their families and attract sexual partners [7, 8].

Traditional healing is a long-established component of the indigenous health system in many parts of Africa, where it is accessed by a large proportion of the population. Preferences among some men to seek care from traditional healers are rooted in conservative gender roles and socialisation processes [6, 9, 10]. Furthermore, traditional healers often have long-standing relationships with their clients, physical exams are not usually required, payment options are flexible, clients are not expected to queue [6, 11] and alternative explanations or diagnoses may be found for HIV-like symptoms, thereby addressing many barriers to health facility attendance. Although a few initiatives have integrated traditional healers into providing key aspects of HIV and TB care, including pre-test counselling, referrals, and adherence support [12], efforts to promote collaboration remain surprisingly limited given that up to 70% of PLHIV in sub-Saharan Africa consult with traditional healers [13]. Greater collaboration between traditional healers and the formal health sector has been heralded as an essential component to end the AIDS pandemic [13–15].

Several calls have been made for innovative approaches to HIV and TB testing that would be more acceptable to men [16, 17]. HIV self-testing has already proved to be effective in increasing diagnoses among previously untested men living with HIV in several African settings [18, 19], but further research is needed to identify new delivery channels [19]. In Eswatini, HIV self-test kits have been offered to patients attending health facilities since 2017 and are also distributed during community events by trained health workers [19, 20], but both these distribution channels may hold greater appeal for women. Given the widespread use of traditional healers in Eswatini and the potential appeal of HIV self-tests to men, we undertook qualitative research to explore attitudes towards integrating traditional healers into the distribution of HIV self-testing kits and containers for TB sputum in order to facilitate diagnoses of both infections.

## Methods

### Study setting

This qualitative study took place between September 2019 and February 2020 in the Shiselweni region of Eswatini, where Mèdecins sans Frontieres (MSF) has supported the Ministry of Health in providing decentralized HIV and TB care since 2007. The predominantly rural region of Shiselweni has approximately 210,000 inhabitants with an estimated HIV prevalence of 26% among adults, corresponding to around 36,000 PLHIV [2]. There are approximately 200 registered traditional healers located in the fourteen inkhundla (administrative sub-division) within the region, of whom around 10% are women.

### Recruitment and training of traditional healers

Traditional healers were identified with the help of the Shiselweni Traditional Healers Focal Point, the head traditional healer in the region, and invited to participate in the study. Enrolment criteria were being resident in the inkhundla closest to the health facility (n ~ 100), expecting to receive clients throughout the study period, a willingness to provide HIV self-tests, wanting to learn more about HIV, self-testing and TB, an intended commitment to the study for six months and no objections to biomedical approaches to HIV and TB care. Recruitment continued until eight traditional healers had agreed to participate as this was deemed the optimal number for the training sessions.

The traditional healers attended a week-long training session which covered HIV and TB risks and modes of transmission, HIV and TB interventions, the use of HIV self-testing kits, as well as how to produce sputum samples. An assessment was conducted at the end of the training which all participants completed satisfactorily, after which the trained traditional healers were each provided with 50 HIV self-testing kits and 20 sputum containers. Each healer was also provided with a log-book in which to record data on the sex of the clients to whom they provided HIV self-tests and/or sputum containers, the number of HIV self-testing kits provided, whether the kit was used in the presence of the healer, and the date of the distribution. Although the intervention was primarily intended to reduce the diagnosis gaps among men living with HIV or tuberculosis, the traditional healers were encouraged to deliver the intervention to any man or women who could benefit. Mobile phone airtime was provided to each traditional healer so that they could stay in touch with their clients and the study team.

### Qualitative data generation

Qualitative data were generated with traditional healers, their clients and health workers through focus group discussions and in-depth interviews (Table 1).

**Table 1:** Participant characteristics per data generation method

Data collection method	Participant characteristics	Number of interviews/sessions
FGD traditional healers	8 men	1 prior to training
FGD community members	5 groups with men, 2 groups with women; 6 people per group	FGDs held throughout the intervention
IDI with traditional healers	8 male TH	8 TH interviewed twice at 2–3 months after intervention start, and 3 months later
IDI with traditional healers’ clients	6 men, 4 women	1 interview per person after receiving an HIV self-testing kit and/or TB sputum containers
IDI with health workers	3 men, 2 women (3 head nurses, two nurses)	1 interview per person during the intervention

A focus group discussion (FGD) was held with the traditional healers prior to the training in order to ascertain their attitudes and knowledge regarding HIV and TB including HIV self-testing and sputum collection, and their perceptions about the intervention.

Individual in-depth interviews (IDIs) were then conducted with the traditional healers at two time-points: between two and three months after the start of the intervention, and three months after the first interview. Repeated in-depth interviews were used to build rapport and gain insight into changes in interviewees’ accounts over time and to examine other layers of narrative beyond those which may be considered socially desirable. Interviews lasted approximately 45 min and were based on topic guides which covered the healers’ attitudes towards, and experiences with, the intervention and any challenges that they faced.

IDIs were also conducted with ten clients of the participating healers using a purposive sampling approach [21] to ensure diversity in terms of their age and traditional healer consulted. Clients were initially provided with general information about the study by their traditional healer. Those agreeing to be contacted by an interviewer were then followed up by telephone to receive further information about the study and to arrange a private place and time of their convenience for the interview to take place. The topic guides covered their attitudes to health-seeking in general as well as for HIV and TB services, including their views concerning the provision of HIV self-testing kits and sputum sample collection provided by the traditional healers.

Personnel in the health facilities serving the same populations as the traditional healers were informed about the study prior to its start by the MSF Community Nurse Activity Manager. Five health workers from these health facilities were invited among which no refusals were documented, with interviews occurring approximately four months after the start of the intervention to assess their views and attitudes towards traditional healers’ involvement in HIV and TB diagnoses. The health workers were recruited by the MSF Community Nurse Activity Manager and were purposively sampled to include different cadres. Interviews were conducted when the health facilities were not busy, and in a private room.

Five FGDs were held with men and two with women from the local community, with six participants per group. The FGDs aimed to elicit social norms relating to traditional healing, health-seeking behaviours including for HIV and TB testing and attitudes towards the intervention.

All interviews and group discussions were conducted by trained Swazi research assistants in SiSwati, matched by sex to the participants who were not previously known to them. Debriefing sessions were held among the researchers after each interview and discussion.

### Data analysis

Interviews were audio-recorded with permission and translated by the research assistants into English. Notes taken during debriefings after interviews were also used in the analysis.

Data collection and analysis followed an iterative process and were conducted in parallel so that topic guides could be adapted if new themes emerged and to enable data collection with clients, community members and health workers to continue until saturation was achieved [21]. Data were analysed thematically, starting with iterative coding undertaken by two members of the research team (BS and NM) to ensure for consistency, with a sample reviewed by EM, with the data organised into topic areas or “nodes”, aided by Nvivo11. Based on principles of grounded theory [21], the codes were regrouped under themes that emerged through the analysis process undertaken by BS and reviewed by EM, with the findings gradually raised to a conceptual level.

### Ethics

The study was approved by the Eswatini Scientific and Ethics Committee (SHR154/2019) and the MSF Ethics Review Board (ID: 1930). Informed consent was obtained from all participants and all methods were performed in accordance with the relevant guidelines and regulations.

## Results

Each of the eight traditional healers received 50 HIV self-testing kits. During the six-month study period, a total of 99 clients consulted the traditional healers of whom 81 (82%) accepted an HIV self-testing kit. The number of kits distributed per traditional healer ranged from 2 to 27. Approximately half of all kits were provided to men (40 (49%), while for 2 (2%) no sex was recorded) (Table 2). Eleven recipients reported a reactive test result for HIV, corresponding to 14% of the tests distributed (Table 2). Each of the eight traditional healers also received 20 sputum containers, of which only 24 of the 160 (15%) were distributed (10 to women, 11 to men and 3 no sex recorded), representing 24% of clients. Six of the healers distributed between 1 and 7 sample containers, while two healers distributed no containers. In total, 20 sputum samples were produced. None of the sputum samples were tested for TB as the health centres did not accept the samples and so these individuals were required to provide further samples for testing.

**Table 2:** Distribution and uptake of HIV self-testing kits and sputum containers

	Number (percentage) of HIV self-test kits	Number (percentage) of TB sputum containers
Total provided to TH	400	160
Distributed to 99 clients	81 (82%)	24 (25%)
Of which female	39 (48%)	10 (42%)
Male	40 (49%)	11 (46%)
No sex recorded	2 (2%)	3 (13%)
Reactive	11 (14%)	_

### Attitudes towards collaboration between the traditional and biomedical health sectors

Prior to the start of the intervention, the traditional healers in the study were highly motivated to be involved in the distribution of HIV self-tests and sputum containers and felt that it aligned with their desire to be recognised by the government and society as legitimate healthcare providers. They also indicated that training and the possibility of “working together” with the biomedical sector would empower them to better help their people:

*Here is something that we are going to learn. That you know, the nation is dying this much, what is killing them, so we are going to hear that…maybe it means when we are working together here, things are going to be fine. (TH FGD)*

Many men in the community also perceived advantages of greater collaboration between traditional healers and the formal health sector, given that traditional healers were occasionally a first step in treatment-seeking. Some participants mentioned that certain ailments could only be treated traditionally and others only by “western medicine”, but also highlighted the potential tensions between providers from each of the sectors:

*I would like to add that this is a good thing for traditional healers to work with the other doctors and they should not look down upon each other…traditional illnesses be treated traditionally and what has to be treated at the hospital be treated there. (FGD men 30 – 45 years)*

Some clients of traditional healers additionally appealed for greater mutual collaboration, respect and referral practices between the sectors:

*in the past, once you came to the clinic with small cuts from the traditional healer [after kugata a form of traditional vaccination done with razors and herbal remedies] they would question how you got those cuts and it would mean you wear something that would hide the cuts because modern doctors didn’t approve of that…and they should also be able to refer you to a traditional healer if they can see that they cannot help you instead of keeping you at the clinic and feeding you with panado [painkillers] (male client of TH)*

Nevertheless, some community members felt that it was shameful to be seen consulting traditional healers, suggesting ongoing stigmatisation associated with seeking services from this sector that could potentially undermine the intervention:

*The truth is, in the community, traditional healers are not viewed well. The proof of that is that people most of the time they do not go to the traditional healers…it is very rare that they go to the traditional healers during the day, they like when it is a bit dark …it is like it is a shame during these times to be seen going to a traditional healer. (FGD men 45+ years)*

Stigmatising attitudes were manifest through beliefs expressed by some community members that traditional healers could bewitch sputum samples that they collected:

*He might “work” on the sputum by bewitching it because maybe he is fighting my family or he is fighting myself...you’ll find that he can do a lot of things with my sputum. (FGD men 19 – 30)*

Attitudes towards traditional healers were also mixed among healthcare workers, with some expressing their expectation that it would be possible to collaborate with traditional healers and that they would appreciate being integrated into the HIV response:

*It can work…it will work every person wants to be involved in something and every person wants to be recognised, it is like you are honouring them actually when you give them such a thing…they'll be like we are being recognised…you know…it will work definitely. (HCW female)*

Such attitudes were not uniform among health workers, with some being disparaging about the idea of collaborating with traditional healers as they did not see them as a legitimate source of providing biomedical health services, with these attitudes often reflecting religious views which were perceived to be in contradiction to those favoured by traditional healers:

*I do not know. Let me say me personally I do not believe in it. Okay, what I forgot at first, when I was introducing myself, was that I am Christian. So, I believe that what the pill fails to treat, the prayer will, that is my belief not anybody else’s. It’s what I believe in: if the pills fail, God is the one to see it, I cannot go elsewhere (HCW male)*

These attitudes partially explained the refusal of health workers in this study to accept any of the sputum samples collected by traditional healers, alongside the perception that they had likely not been obtained using suitably rigorous procedures.

### Perceived advantages of seeking TB and HIV services from traditional healers

Several male clients of traditional healers expressed their preference for accessing tests from traditional healers, rather than going to the hospital. In contrast to health facility testing, many participants considered that going to see a traditional healer offered greater privacy, thereby helping to overcome this barrier to testing uptake:

*In my opinion, this can work because men are happy to go to a traditional healer. There’s privacy and they are alone with the traditional healer. No matter what problem that person has, no one knows why they had visited a traditional healer, it is easy for the people. (TH 2)*

Some traditional healers reported how their clients expressed relief and wonderment about now being able to access an HIV test from a TH, rather than through formal health facilities, suggesting a general acceptance among clients in accessing HIV testing from a less exposed environment:

*Wherever I go, they want the kits…they are happy because they are getting it from their hide-out which is the traditional healers. They are happy that we are now working with medical doctors and they wonder how is that happening. And then I tell them that shouldn’t bother them, but instead they should just take the kits and that’s all. (TH 2)*

Furthermore, in addition to offering HIV tests, some male traditional healers were able to engage with their clients on topics including HIV risk reduction and prevention, or adherence to antiretroviral therapy, demonstrating their potential as effective “man-to-man” peer educators:

*My cousin is someone who I talk to when I have any kind of a problem, so he told me to go and test with the traditional healer. I said: “No! I am not going there”. He went and then he came back and asked me and then I said: “ok I will go”. I asked the traditional healer if he would advise me to take the pills and where to go for it. ,… and then he said: “It is life, you will not die. It depends on you and how you are taking care of yourself, what I am doing like with girls and those razors [traditional medicine “injected” through small cuts] and using all those things and touching blood. So I listened to him. After two months, I went back there: I was then ready to go and test, so I tested. (male client)*

Frank talking by traditional healers to their clients was also seen as being important in encouraging men to take up self-testing kits:

*It is begging them! Really propose them straight, just like when proposing love to a lady, so that the person can accept the box [HIV self-tests] and go to test themselves. Besides, if you are not able to talk to people well, they are really not going to accept them. You need to be able to talk to people for them to accept the box and go to test themselves. (TH 6)*

Traditional healers who had experienced tuberculosis themselves could relate to the illness experiences of their clients, and some also had first-hand knowledge of seeking formal health services which they could draw on in order to convince their clients to test and to take treatment:

*For me, I suffered from TB. I was very resistant going to the hospital and then I felt that oh, actually now I am pulling with my last gear! My wife took me to the hospital, and at the hospital they found TB, and they gave me pills that I took for 6 months, and I was fine until today. (TH 6)*

Despite the general acceptability among male clients of traditional healers in receiving an HIV self-test kit from them, there were still some persistent concerns about confidentiality which could undermine its uptake:

*They [men] do not trust you, they just think you will go around talking, just like these other people here. They took [test kits] and went. Just as I say it is going slowly, it is because this one is not finished yet [HIV/TB and stigma], But those who are now educated, they are testing, they test freely, I do not want to hear people saying I came here and tested, it is that. (TH 5)*

## Discussion

This study used qualitative methods to investigate attitudes towards and experiences of integrating traditional healers into the distribution of HIV self-tests and sputum containers in a rural setting in Eswatini.

Our findings suggest that the traditional healers were able to distribute HIV self-testing kits and sputum containers to some of their clients. However, while the HIV self-tests resulted in an important proportion of reactive test results, our study design was unable to determine the proportion of these that were first diagnoses, an issue which merits further investigation. We found that the provision of HIV self-testing kits by traditional healers was valued by many of their clients as it afforded greater privacy in the context of ongoing and pervasive stigmatisation of HIV that can be experienced when attending health facility settings [21, 22]. Nevertheless, it was also apparent that seeking care from traditional healers was often a stigmatising behaviour, with derogatory attitudes reported among some health workers and community members. In the context of appeals from traditional healers and their clients for greater collaboration between the sectors to enable the provision of holistic care for Swazis, we recommend that further efforts are made to promote the dialogue, cooperation and effective referral practices between them. In relation to HIV self-testing, specific interventions should be developed to ensure that clients with a reactive test result are effectively linked to confirmatory testing and antiretroviral therapy.

Our findings also showed that sputum samples were not accepted by health centres in the study area, which may reflect insufficient preparation of the health workers to receive the samples as well as tensions between the world views held by some traditional healers and health workers. Additional research is needed to further investigate and address this barrier to this TB case-finding approach if this intervention is to realise its potential to diagnose “missing cases” of TB.

Identifying acceptable modes of delivery for HIV and TB services for undiagnosed men remains a challenge with its roots in health facility-based responses, including through integration of HIV care in reproductive and sexual health services that are designed to serve and prioritise the needs of women. Furthermore, the negative effects of men’s socialisation on their own health and in fostering their reluctance to use health services has been well documented, including in studies that underline men’s agency in constructing masculine ideals and powerful patriarchies that place them in disadvantaged positions with regards to their well-being [5, 6, 8, 14, 23]. In a context where norms around masculinity and sexuality undermine the willingness of men to seek health facility-based services, the provision of HIV self-testing kits through traditional healers represents a pragmatic solution to the relatively lower rates of HIV diagnoses among men. Specifically, we found that men often felt more comfortable in discussing TB, HIV and sexuality with male peers from the same community, which were often based on long-standing relationships. Traditional healers demonstrated cultural literacy through their ability to engage with the logic and beliefs of their clients [12]. These findings chime with those from Ndlovu who found that traditional healers in Eswatini play a moderate but meaningful advisory role in supporting current biomedical HIV and TB strategies by fostering behavioural change amongst their clients [24]. Regardless of the advances that are made in terms of biomedical innovations, coverage of TB and HIV interventions is likely to remain sub-optimal unless greater efforts are made to understand how cultures and social norms influence HIV and TB health-seeking behaviours and ensure that health systems respond to individual needs and preferences as expressed by communities and individuals affected by HIV and TB [5, 25].

Our findings need to be considered in light of certain methodological limitations, including the potential for social desirability in participants’ responses, despite field worker training. Furthermore, some of the healers found it challenging to complete the logbooks that were provided, and in a bid to simplify record-keeping, no data were collected on the characteristics of clients who did not agree to take an HIVST or TB sputum sample container, despite being offered one, nor on previous testing histories or subsequent linkage to care for clients with a reactive HIV self-test. Finally, the study was undertaken in an area where MSF has been supporting the Ministry of Health to provide HIV and TB services and thus the exposure of the population to these services may differ from other areas of the country.

In conclusion, our study demonstrated that distribution of HIV self-tests via traditional healers is likely to reach some men who may be missed through health facility-based HIV testing models or other HIV self-test distribution channels. In the context of Covid-19 in Eswatini, the provision of HIV self-tests through a range of channels has become even more important as a means to mitigate the impact of the pandemic on the uptake of HIV and TB services in health facilities.

The intervention was appreciated by the majority of healers’ clients who took up the offer of HIVST or sputum collection containers, due to the cultural literacy of traditional healers and practical considerations. Scaling-up this approach could bridge HIV testing gaps, particularly in the context of Covid-19, if traditional healers are well supported, but further research is needed to understand barriers to uptake, and to strengthen procedures for receiving sputum samples at health facilities to increase TB diagnoses.

## Data Availability

All data generated or analysed during this study may be obtained via the corresponding author upon request.
